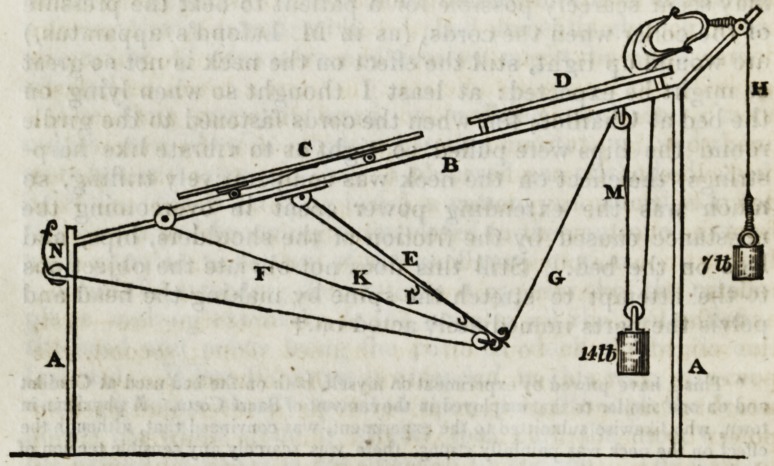# An Account of the Mode of Treating Distortions Adopted in Paris; with Remarks

**Published:** 1826-12

**Authors:** John Shaw

**Affiliations:** Surgeon to the Middlesex Hospital.


THE LONDON
### Medical and Physical Journal.

N° 334, VOL LVI.] DECEMBER, 1826. [N° 6, *New Series*.

For many fortunate discoveries in medicine, and for the detection of numerous errors, the world is indebted to the rapid circulation of Monthly Journals; and there never existed any work, to which the Faculty, in Europe and America, were under deeper obligations, than to the Medical and Physical Journal of London, now forming a long, but an invaluable, series.—RUSH.

### ORIGINAL PAPERS, AND CASES OBTAINED FROM PUBLIC INSTITUTIONS AND OTHER AUTHENTIC SOURCES.

### DISTORTIONS OF THE SPINE.

*An Account of the Mode of treating Distortions adopted in Paris; with Remarks.* By JOHN SHAW, Surgeon to the MIDDLESEX HOSPITAL.

As the spine is composed of many parts, which differ essentially from each other both in structure and function, it is liable to a variety of diseases. On a former occasion* I proved this to a certain extent, by the description of nearly an hundred morbid specimens from the private collection of Mr. BELL. But we need not refer to a museum of morbid anatomy for our proofs: any surgeon in extensive practice will admit that there is much variety in the diseases of the spine, and that the symptoms are so varied, so curious and complicated, that he can scarcely enumerate them. In the Middlesex hospital, at this moment, there are seven patients with affections of the spine, and there are not two in whom the symptoms are similar, or where the same mode of practice is applicable. But, notwithstanding the difficulty which the highest medical authorities have in determining on their nature, although a mistake might prove fatal to the patient, these complaints are frequently entrusted to empirics or to mere machinists.

Although men ignorant of anatomy and pathology, and proceeding merely on mechanical principles, are dangerous practitioners, yet, in one of the most common affections of he spine, little good can be done without the aid of mechanical

* See the Appendix to my work on Distortions of the Spine, and the volume of Plates in illustration of the varieties.

means; I have therefore taken every opportunity of examining the various contrivances which have been proposed, either by medical men or by machinists, in expectation of receiving hints for improving and simplifying one of our most useful auxiliaries in the treatment of these diseases.

Having repeatedly heard of some extraordinary methods practised in the institutions lately established in France for the cure of distortions, I went to Paris last August, in the hope of being able to see them. Through the kindness of my friend M. Breschet, surgeon of the Hôtel Dieu, and professor in the Ecole de Medicine, I was introduced to the medical superintendents of several of these institutions. These gentlemen shewed me their method of treatment, and, fortunately for my object, the patients at each of the establishments cheerfully permitted me to examine them, and answered my questions with the greatest readiness. In this way I had the fullest opportunity of learning each plan of treatment.

There are many establishments in Paris for the cure of distorted spines: I visited three of the most celebrated. The principal one is in the Rue Varenne, in one wing of the splendid building formerly the Hôtel Biron; the rest of the house being occupied by *pensionnaires*, who are educated by the nuns of the order of Sacré Cœur, the institution is now called "the Convent of Sacré Cœur."*

I also visited the institution superintended by Dr. Maisonabbe, the editor of a journal devoted to the discussion of questions connected with the subject of distortion. His institution is in the Rue Chèvreuse, near the Luxemburg. I also saw many patients in the establishment superintended by MM. Lafond and Duval, in the Rue de Batailles, at Chaillot, one of the most beautiful rising grounds near Paris.

As I had thus the advantage of seeing the different modes in which about seventy patients† were treated, I made no attempt to see the institution at Chaillot superintended by M. Milli, although his was the first established in Paris, and is perhaps more talked of than any other. The history of its foundation is rather curious. M. Milli was a mer-

* Dr. Recamier of the Hôtel Dieu, and Dr. Collin of la Charité, are the medical superintendents: the *traitement* is superintended by a governess, who had formerly the charge of some young ladies who were patients in M. Milli's institution at Chaillot, which will be described presently.

† Some idea of the rank of the patients treated in one of the institutions I visited may be formed, from each of them paying 6000 francs a-year: there were thirty young ladies there.

chant:* when twenty-three years of age, he consulted his family physician about a curve in his spine. The physician recommended him to M. D'Yvernois, then famed in Paris for curing club-feet; but the latter not choosing to undertake the treatment of a curved spine, advised him to go to M. Heine, at Wurtzburg in Germany, there not being at that time (1820) any institution in Paris for such cases. M. Milli remained for some time at Wurtzburg, and, while there made drawings and models of the beds and other contrivances used in M. Heine's institution. On his return to Paris, he called on his friend the physician, and assured him that he had received great benefit from the plan pursued at Wurtzburg; but the physician declared, on examining him, that he saw little change in the state of his spine.† However, he complied with M. Milli's request, and certified that the beds, &c., of which M. Milli shewed him the models, might be useful in the treatment of curvatures of the spine. M. Milli received similar certificates from other medical men in Paris. He then published a Prospectus, in which he proclaimed that he was cured, and promised, by following a similar plan to that by which he had been relieved, to cure all diseases of the spine in an establishment he had formed at Paris, in the Quai de Billi, near the Champs Elysées.‡ This was so lately as the winter of 1823. The speculation succeeded so well, that there were several institutions immediately established on the same plan, and even some mistresses of boarding-schools, finding they were losing their pupils, got beds made similar to those used by the young merchant, and took the treatment of the crooked into their own hands. It is stated in one of the French Journals, that, of 140 young ladies in one school, twenty were found to require treatment.

* See the Journal Clinique, par C. A. Maisonabbe; Professeur Agregé en Exercise a la Faculté de Medecine de Paris; and La Verité sur les Progres recens de l'Orthopedie, par un Docteur en Medecine de la Faculté de Paris.

† See the communication from this physician to M. Maisonabbe, in the first Number of the Journal Clinique.

‡ A son retour, M. Milli s'empressa de faire construire un lit semblable a ceux de Wurtzburg, et s'étant procuré l'attestation de quelques medecins et chirurgiens, recommandables qui crurent vraisablement, que les lits mécaniques pouvaient contribuer à corriger les courbures de la colonne vertebrale, il publia un prospectus anonyme dans lequel, il proclama hautement sa guerison, (quoi qu'il ait encore assure-t'on besoin de corset pour dissimuler l'inegalité des ses épaules,) et la promit, sans scrupule, aux infirmes qui voudraient se rendre dans un établissement qu'il venait de fonder a Paris, Quai de Billi, près les Champs Elysées. (Hiver de 1823.)—See p. 5 of "La Verité sur les Progres recens de l'Orthopedie," par un Docteur en Medecine de la Faculté de Paris.

M. HEINE, of Wurtzburg, in a paper on the instruments used in the Treatment of Distortions, (in the Jena, Allge. Literat. Zeitung, for Dec. 1825,) complains bitterly of M. Milli's conduct, and declares his disbelief in the announcement of a pretended cure. M. Heine says that the apparatus which M. Milli brought from Wurtzburg, however perfect in itself, is not sufficient for the cure of a person twenty-three years of age; and that, to cure any curved spine, a long and complicated treatment is necessary, &c. &c. However, although this young merchant has many enemies and rivals, he has also powerful friends; for some of the members of the royal family have occasionally visited his institution, and given their *sovereign* opinion that the method he pursues is very judicious. He has also a strong ally in the Constitutionel newspaper, which often contains paragraphs in favour of his establishment.

It may appear strange that patients labouring under a very serious and complicated disease, should be entrusted to the care of a man entirely ignorant of medical science; but so many were attracted by the novelty of the plan, that other persons were induced to found rival institutions, where the same mode of practice was followed. Indeed, as there were no similar institutions in Paris previously to M. Milli's return from Wurtzburg, this person, although not educated to the profession of medicine, but acting as a merchant, may be considered as the founder of the establishments which, within the last two years, have become so numerous in Paris and in the provinces of France. We may express surprise at the credulity of the French; but when we recollect the history and the temporary success of some adventurers who proposed to cure similar diseases in London, we have no right to call ourselves a more "thinking people" than our neighbours.*

As M. Milli avows that he has copied from M. Heine, we should, without going further into the history, be led to suppose that M. Heine was the original inventor of the mechanical beds, &c.; but we shall find that Heine's method is nearly a copy of that used by VENEL, who practised about

* The frequent announcements in the French papers, and even the "Rapports" of the Faculty of Medicine, give some idea of the variety of inventions in Paris at this moment for the cure of distortions. Any one who is curious to see one of the last improvements, may perhaps have an opportunity by calling at an upholsterer's in Mount-street, where there is "un lit mécanique." It was invented by a locksmith in Paris, and, after being presented to the Academy as being superior to all prior inventions, was sent over here; the maker expecting to receive a large sum for the right of using it.

fifty years ago at Orbe in Switzerland, and which is described in the Memoirs of the Physical Society of Lausanne. It is there stated that Venel used, during the day, a machine nearly similar to Levacher's, or to that known in this country by the name of Jones's collar; but that his grand improvement on Levacher's system was a contrivance for fixing the patients in a state of extension during the night. The Vth plate in the 2nd vol. of the Memoirs represents the bed employed by Venel, and, if compared with that now generally used in Paris, and which is called the Wurtzburg bed, the difference between the two will be found so trifling, that we shall be inclined to agree with those who allege that Heine, who complains of Milli, is himself a mere copier of Venel.

Before making any remarks on the principle of the practice followed in the several institutions, I shall endeavour to describe the methods by which I saw it put into effect.

In the convent at Sacré Cœur, the beds are formed after the model of those used by Milli, or after that of Heine of Wurtzburg. The sketch is on so small a scale, that it is difficult to represent accurately the form of the springs. In the last improvements by Martin, the machinist of the Institution, there is a scale introduced upon which the elliptic springs act: he has also added a windlass, or wheel, at the lower end of the bed, to act on the springs. The springs are very powerful, and, by communicating with the casque on the head and the girdle round the pelvis, keep the body on the stretch. The bed or mattress is stuffed very hard, and is so convex that the spine, or only the middle of the back, is supported: indeed, it is of such a shape that it would be impossible to remain on it if the body were not fixed. Several springs (not represented in the plan) are attached to the sides of the bed: some project in a direction nearly horizontal above the mattress, so that the sides of the patient, instead of resting on the bed, are supported by several points. The object is to push in the projecting parts: while other springs pass over the body, and press upon the ribs in front. The patient spends the night, and the greater part of the day, on this bed; a constant pull being kept on the head and the hips by the elliptic springs, while pressure is made on the protuberances by the horizontal *plaques*. When she rises from bed, she is put into a strong arm-chair, furnished with a *minerve* and powerful springs, which may be brought forward by screws. Placed in the chair, she is first firmly strapped down, by a girdle round the hips, to the seat; her head is then elevated, and fixed to the minerve; then the springs are worked forward by screws to press on the ribs: she has thus no power to move her body in any direction. When freed from the chair, she either puts on a strong corset or cuirass, also furnished with strong-pressure springs; or she mounts on long crutches, which prevent her touching the ground except with the tips of her toes. It is only on such crutches that she is allowed to stand or move about. When sitting at her meals, she is supported by crutches attached to the chair. I cannot state the exact time spent in each of the machines: I saw all of them used, with the exception of the corset, and was told that there was not an hour, night or day, during which the patient was not engaged in one or other of the plans of treatment.*

That this description is not exaggerated, will appear by the following extract, from the Journal Complémentaire des Sciences Medicales for May 1824, of a description by Professor Foderé of the method pursued in an institution lately established by M. Humbert:

"Each patient was put into a separate bed. The placing them occupied us from nine until one in the morning: (there were twenty patients.) The beds had pulleys at the sides, and levers at both ends. Each patient, covered with a flannel gown, which opened behind, was laid on a hair-quilt, four inches thick, and without cushions or bolster. A large leathern belt, with rings for attaching the several cords, was put round the hips; and upon the head a cap, laced on the upper part, and fastened under the chin. This cap was attached to a long lever at the top of the bed. The patient

* All the machinery is beautifully executed and finely polished. The maker is Martin, who, like our machinists in London, also undertakes to cure diseases of the spine. As long as complicated machinery is recommended for the cure of distortions, such persons will generally become the advisers, instead of continuing to be merely the manufacturers of the instruments ordered.

being now laid at full length, the operator exposed the protuberance *(bosse)*, worked it and kneaded it *(la masse et la petrit)*, while he pushed it from the opposite side. He then insinuated a wedge of wood, prepared according to the form of the tumor, between it and the mattress, so as to push the swelling inward. If the curve was the shape of an S, this operation was done on both sides. On the side opposite the tumor, which is generally thin and wasted, dry friction, and even slight "*flagellations,*" were used to excite the contractility of the muscles. After these processes were completed, the body was, by cords passing from the girdle round the hips, fixed to the sides and ends of the bed, so as forcibly to pull down the trunk and pelvis from the head, which continued attached by the cap to the lever at the top of the bed. "Cela fait, on souhaite la bon soir a la patiente, et l'on passe à une autre."

"At four in the morning, a new order of operations commenced. Each patient in rotation was placed in a vapour bath, where she remained an hour; after which her *bosse* was exposed to a water vapour douche *(de vapeur aqueuse)* for half an hour, it being *massé et petrit* at the same time, and the opposite side slightly *flagellé.* She was then taken to her chamber, and placed in a mechanical arm-chair, made on purpose for herself; a desk was added to it, to support her breakfast and writing or drawing apparatus, as she was to remain there four or five hours, without moving any part, except her limbs; the chair being so contrived that the wedge called *debossoir,* and the continued extension of the neck and trunk, could be employed as in bed. The patient thus did not enjoy more than seven or eight hours of liberty: if, indeed, that can be called liberty where she was not allowed to quit her long crutches."

The Professor continues:—"I confess that, in the three or four first instances, I was quite overpowered at this species of torture, but I was soon able to bear the sight unmoved; for, during all these operations, the patients did not even change colour, and each, in reply to the questions I put, *avec l'air de m'appitoyer sur elle,* said, smilingly, she did not suffer; that she slept very well, although tied up; and was so accustomed to the wedge, that she felt a want when it was not applied. I also examined the pulse and respiration, but there was no change in them: 'tant est puissant chez les femmes le désir de paraître belles.'"

The diagram in the next page gives a general idea of the mechanism of the bed used by M. Maisonabbe. The extension of the spine is produced by an ingenious, although rather complicated apparatus, but by it M. Maisonabbe says he can regulate the degree of power more easily than by the springs used in the beds at Chaillot, and at the convent of Sacré Cour. As the manner in which the patient is laid and fixed is sufficiently explained by the drawing, I shall describe only the diagram of the machinery. In the bed the machinery is all concealed by a board; there is only a hole communicating with the axle of the wheel E, and a scale with a pointer, seen on the outside of this board. A, is a piece of wood passing from one side of the bed to the other; BN, BN, are two boards, twenty-eight inches long and four broad, attached to it by hinges; their moveable ends are fastened to the cords coming from D and H. P, P, are weights, of twenty-five or thirty pounds, mounted on wheels, and running in grooves on BN: these weights are attached by cords to the wheel E, which may be turned by a key. As the weights are raised or depressed on B, N, the power of these levers is increased or diminished. The cord attached to the collar round the neck is graduated. When the patient is to be submitted to the extension, she is first fixed by the collar G round the neck, and by the circle H round the hips, to the cords passing through the upper and lower ends of the bed. The operator then stands behind the upper end of the bed, and, taking hold of the patient's head, extends the vertebræ as much as he thinks proper, by pressing with his foot on the lever at N; then, by turning the wheel, arranges the position of the weights, and, noting their effect on the graduated cord, contrives that the continued extension shall be less by one-half than that produced by pulling with the hand and pressing with the foot.

As far as regards the mode of effecting extension, this is better than by springs, since more elasticity and play are permitted. M. Maisonabbe does not apply pressure, but trusts to the effects of the extension of the spine. His patients also use the long crutches in walking, and the crutches attached to the chair while sitting at table.

In the first Plate, which represents the bed and apparatus used by MM. Lafond and Duval at Chaillot, the machinery appears very complicated. This is from the inventor conceiving that a permanent extension ought not to be made on the spine, but that there should be what he calls an oscillatory movement of the different parts of the column; that they should pass alternately from a state of rest to a state of extension. The patient being fixed in the manner represented in the plate, is first wound up to a certain degree of tension by a ratch wheel, to which the cord seen passing over the pulley at the foot of the bed is attached: when thus stretched, an eccentric wheel (over which the extending cord passes,) is put into motion, so that the extension is increased, and then returns to its former degree. The effect of this apparatus in producing an oscillatory motion, may be understood by the following diagrams:

*Fig.* 1.

*Fig.* 2.

Let A, fig. 1, be a cord fixed at E, passing over a circular wheel B, and over the pully D, and supporting the weight C. When the wheel B revolves, the position of C continues the same; but if B, as in fig. 2, be made of the form of an eccentric wheel, when B is turned, C will rise or fall according as the long or short diameter of B is uppermost.

The eccentric wheel was intended to be kept in constant motion by the mechanism at the end of the bed, but this machinery was not in use when I visited the institution.*

* So great is the desire at present in Paris to do every thing by machinery, that a proposal was seriously made to erect a steam-engine for the purpose of making the extending forces more equable.

The oscillatory motion was produced by the patient turning a winch connected with a ratch-wheel, which communicated with the eccentric wheel by a chain. The turning of the wheel by the patient herself was supposed to be advantageous, as the right or left arm might be used according to whichever side was defective: but to this alleged advantage I could not assent, as the shoulder-joint only was exercised in doing this.

The inventors allowed me to be fixed in the position represented in the plate, to try the effect of the oscillatory movement. Before I could be made sensible of the tension being increased by the eccentric wheel, I had to request that the small windlass, by which the body is extended in the first instance, should be wound up to its utmost pitch. It appeared to me that, unless the body is at first very forcibly extended, the effect of the eccentric wheel must be slight. Indeed, since the hips rest on the bed, there is scarcely any relaxation, and hence the apparatus is little more than the means of increasing a force already considerable. I shall presently show how an alternation of relaxation and tension might be more easily effected.

The long crutches used in walking, and the chair-crutch, were likewise employed in this institution; and there were hot and vapour baths. I observed one of the beds used by Milli, (the Wurtzburg bed:) a patient was placed in it for the purpose of becoming accustomed to the continued extension, previous to the use of the oscillatory motion.

From the preceding descriptions it appears that the principle in making extension is the same in all the institutions, the head and pelvis being the parts to which the bands are fastened. When the spine is extended in this way, the cervical portion is chiefly operated upon; for, although the force affects the whole spine, it strains that part the most where the extending power is not impeded by friction. The weight of the shoulders, chest, and abdomen, resting on the mattress, cause a great deal of friction; while the neck, not rubbing on the bed, offers no impediment of this kind. It is also to be recollected that the cervical vertebræ are less encumbered by connexions with the adjoining parts than the dorsal or lumbar.

The objections to this mode of extending the spine will appear stronger on reflecting that it rarely happens that the cervical vertebræ require extension; while, in almost every case, an extending power, much stronger than can be borne by the cervical vertebræ, should be applied to the lumbar part.

It may be further observed, that, by the manner the force is applied, there is even more friction to be overcome than that produced by the weight of the body alone,—viz. that of the hips and legs; and still the increased force necessary to do this also operates on the neck. We are therefore justified in concluding that, from the impediments caused by friction having been overlooked, the machinery stretches a part of the spine which scarcely requires it, while it operates to a comparatively trifling extent on that portion which may be most benefited by extension;* and that the force necessary to overcome the friction is so great as to be almost unmanageable.

As the head is not fixed in M. Maisonabbe's bed in the same manner as in those of the convent, the force operating on the neck may not appear so great as that on the loins; but the same objections apply to this mode, because the two extending powers are equal, and the head and the hips are still the points on which the pull is made. However, although it may seem scarcely possible for a patient to bear the pressure of the collar when the cords, (as in M. Lafond's apparatus,) are wound up tight, still the effect on the neck is not so great as might be expected: at least I thought so when lying on the bed at Chaillot; for, when the cords fastened to the girdle round the hips were pulled so tight as to vibrate like harp-strings, the effect on the neck was comparatively trifling, so much was the extending power spent in overcoming the resistance caused by the friction of the shoulders, hips, and legs on the bed. Still this does not obviate the objections to the attempt to stretch the spine by making the head and pelvis the parts immediately acted on.†

* This I have proved by experiment on myself, both on the bed used at Chaillot and on one similar to that employed in the convent of Sacré Cœur. A physician in town, who likewise submitted to the experiment, was convinced that, although the effect on the neck was painfully strong, there was scarcely any sensible tension of the lumbar part.

† Since writing the above, I have had an opportunity of seeing the apparatus used by a young lady, who, for the last eight years, has been trying a variety of plans for the cure of a curvature in her spine. She submitted for the prescribed time to wear a machine similar to that represented in the 7th Plate of my work on Distortions, as an improvement on Chesher's collar; after that, she suffered for some years from the contrivance invented by a machinist in Great Queen-street,—a sketch of which is also given in the same plate; she then had the perseverance to go more than four miles every day, except Sundays, for many months, for the purpose of climbing up ropes and performing similar feats during several hours: but, being disappointed in the result of all these plans, she went to France, and has brought back an apparatus like that used at the convent of Sacré Cœur, determined to give it an eighteen-months' trial. The physician who took me to see the apparatus tried the effect of the extension by the collar on himself: he can best tell what he felt; my sensations were so alarming that, before I was stretched for a minute, I entreated to be released. I can now understand the feelings which cause such distress to patients who complain of fulness in the head; and knowing that the col-

As it is perhaps now obvious that the springs used at the convent of Sacré Cœur, the ratch-wheel at Chaillot, and the weights and levers of M. Maisonabbe, are not adjusted so as to operate on the different parts of the column, I will not enter into a more minute detail of the manner in which these forces act. Perhaps my objections to the various modes of extending the spine, will be more easily understood by comparing the French beds with a moveable plane, which I have been in the habit of recommending. The apparatus is nearly the same as one I contrived about eight years ago, but the drawing given in the folio edition of the Plates represents it as too complicated. At the time the drawing was made, I imagined that the additions were improvements, but I have been since induced to restore the machine to its original simplicity; and therefore, although it is the same in principle, it may appear to be a different instrument from that represented in the plate formerly published.

If this contrivance were merely for the purpose of extending the several parts of the spine, it would have been represented in a much simpler form; but, as it can also be adapted for a variety of exercises, all the cords and pulleys necessary for performing them, have been introduced into the diagram.

lar is *sometimes fixed by a padlock during the night*, I can believe that the worst effects may ensue from this practice. The cause of my feeling the pressure on the neck more on this occasion than at Chaillot, was probably from the head-spring being differently arranged. This might be altered; but, if it be true that the springs are in some instances so managed that the eyes become suffused with blood, we can form some estimate of the danger to the delicate vessels of the brain.

The frame-work is six feet long and twenty inches broad, and is like a small bed with one end higher than the other; the middle space is filled up with thin deal instead of canvass, and there are ledges on the sides to confine the moveable frame. C is thirty-two inches long, stuffed and covered with rough green baize, and mounted on six rollers. D is eighteen inches long, stuffed and covered, and may be fixed temporarily by pins into holes in the large frame. E is a strong door-spring, such as may be got in any ironmonger's shop.

If D were taken off, the apparatus, as represented by the diagram, would be ready for the performance of the various exercises; but I shall suppose that a patient is to be put on it for the purpose of an extension being kept up on the several parts of the spine. D being on, C is brought up close to D, and fixed there by slipping a ring on a brass stud in D. The loop K is to be put on the hook L, and the fourteen pound weight is to be hooked on the loop M. The patient now lies down on D and C: she should have on a dressing gown made of the nappy cloth called Bath coating, as this, to a certain degree, prevents her slipping when the extending force is applied, but it is not sufficient; therefore a broad belt, fastened by straps to D, is buckled round the waist, and another fixed to C is put round the hips. The ring by which C is held close to D is now to be thrown off; C is then pulled down by the spring lever and weight, and draws the hips from the chest, so as to extend the lumbar part of the spine. It must now be obvious that, as the body is carried down on rollers, the extending power will not be impeded by friction, as in the French apparatus. The forces operating are—first, the weight of the body on the inclined plane; second, the power of E as a spring; thirdly, the fourteen pound as a weight; but greater than all is E as a lever, increased in power by the cord being fixed to L, and the fourteen pound to its extremity. It is unnecessary to describe the effect of this arrangement of the lever and weight; it will be sufficient to state that in this way we may have a force equal to sixty pounds; and which, as it separates C from D, necessarily operates on the lumbar part of the spine. This is certainly a great force, and, although it may be reduced in a moment to one of ten pounds, it ought to be used with caution: it is, however, the power with which I have been for some time operating on the lumbar part of the spine of several private patients, and of a girl in Handel's Ward. As the latter patient is seen almost every day by my colleagues and the pupils of the hospital, any bad effects, or even inconveniences, resulting from the force applied would have been immediately observed. It may be said that an apparatus of such power is more dangerous than the beds used by the Germans or French; but there is no useful surgical instrument, nor medicine, which may not be dangerous if put into the hands of one unacquainted with the principles of our science.*

This power, acting on the lumbar part of the spine, does not in the slightest degree affect the cervical vertebræ. When it is necessary to operate on the upper portion of the spine, a new power is employed, and one so simple that it may be regulated with the greatest nicety. A broad soft band is put under the occiput, another under the chin or over the forehead, according to the shape of the patient's head: these are hooked on a *spur*, to which a cord, H, with a weight, is attached. The head, after being drawn up by the operator's hands, so as to extend the cervical part of the spine, is laid on a small pillow on D: a weight of ten pounds will keep the head in its proper position. By following the same principle, we can operate, with different degrees of power, on any portion of the spine.

We may now attend to the apparatus, as the means of performing exercises which tend to increase the power of the several classes of muscles.

By merely lifting off D, taking K from the hook L, and the fourteen pound from M, leaving it attached to its pulley, we have the apparatus as represented in the diagram, and so arranged as to permit of the performance of a great variety of exercises: I shall only mention a few by way of example.—The patient may lie on C, and pull herself up to the top of the plane, by taking hold of pegs which project from the sides of the frame. The spring and weight will pull her down; or she may imitate rowing, by sitting on C, and drawing herself up by a cross stick attached to cords in the upper part of the frame. By standing opposite to the bottom of the plane, she may, by hooking the cross stick to N, imitate the motions of a sawyer; or, by putting a band over her head, bring the long muscles of the spine more immediately into action. The resisting force in all these exercises is the combination of the spring and weight, which may be varied in power according to circumstances. Although the double pulleys diminish the force by one-half, they have been added for the purpose of admitting a greater latitude of motion.

The whole apparatus is so simple in its construction, that it requires no further description. As far as it is the means of

* Of seven patients at present in the hospital with affections of the spine, there is only one for whom this practice is applicable: it would be highly dangerous in the other cases.

extending the spine, it is not liable to the same objections as the French beds. There is no friction; the extending power may be modified, or applied to any particular part of the spine; the cervical may be extended in a slight degree, while a strong force is operating on the lumbar and lower dorsal vertebræ. There are also other advantages: the patient may immediately stop either force or the whole, and be as completely at rest as on a common inclined plane; or she may, by taking hold of pegs at the top of the plane, relieve herself by alternating the stretching power with exercise; and, as the power is always acting, were it desirable, the oscillatory motion might, by the introduction of two wheels, be made more complete than that produced by the complicated machinery of M. Lafond.

But, independently of its being an apparatus for extending the different parts of the spine, it admits of the patient taking such a variety of powerful or gentle exercises as to obviate the necessity of any other contrivance for bringing the muscles into that state of excitement which is so important in the treatment of distortions.

Since it is acknowledged that little good can be done in many cases of surgery without the aid of mechanism, and that much harm ensues from its misapplication, it is to be hoped that the subject of this paper, although almost purely mechanical, will not be considered unworthy of attention. It is surely important to inquire into the fitness of new contrivances for the treatment of a disease, which, in many of its stages, may be greatly benefited or aggravated by machinery; and especially at present, as the system now pursued on the continent will probably soon be introduced into England.*

Many questions regarding the principles on which the German system of practice has been adopted in France, remain for discussion. The mechanism of the beds as a means of extension has been first examined, as it is the part of the system to which the French seem to attach the greatest importance. We have still to inquire into the propriety of the use of pressure, into the advantages of the long crutches, and into the cause of the apparent neglect of all those gymnastic exercises which were at one time so much in vogue in Paris.† But the consideration of these and other questions connected with this subject, must be deferred until another opportunity.

* Several young English ladies were in the institutions in Paris when I visited them; and, since my return, I have heard of fifteen who have gone there to submit to the treatment, and of several who have returned.

† The climbing ropes, &c. for the cure of distortions, although imported from Paris to London, is now entirely given up there: it has, like every other system

## Figures and Tables

**Figure f1:**
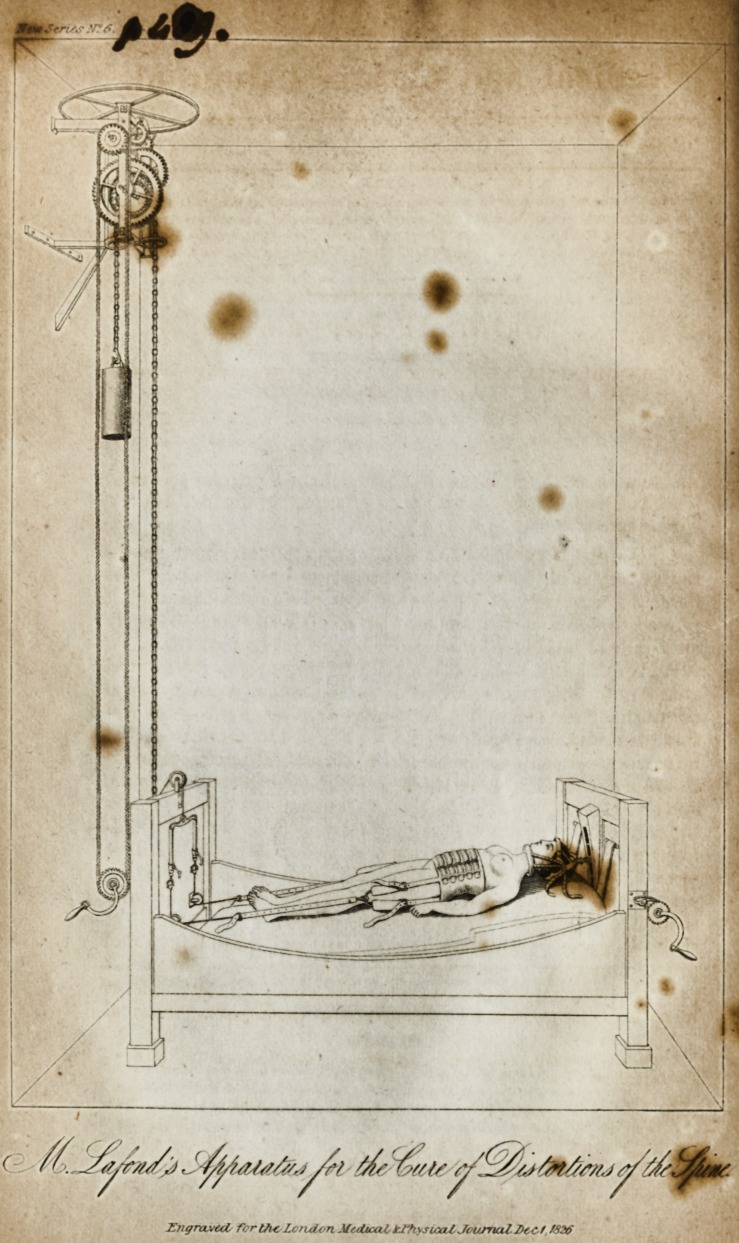


**Figure f2:**
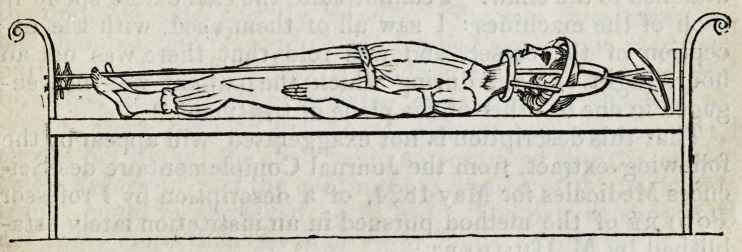


**Figure f3:**
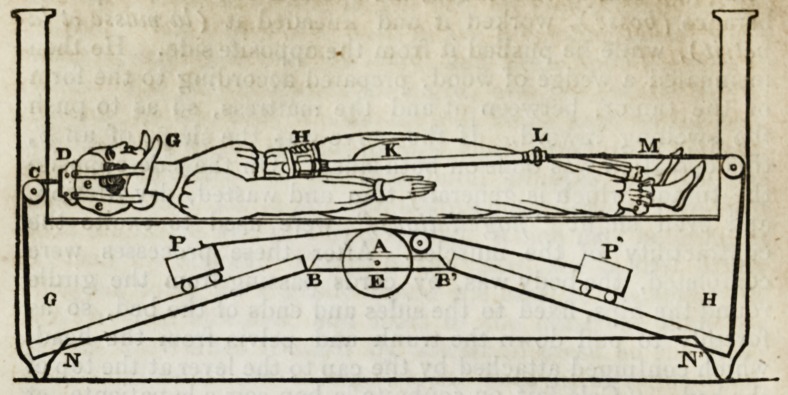


**Fig. 1. f4:**
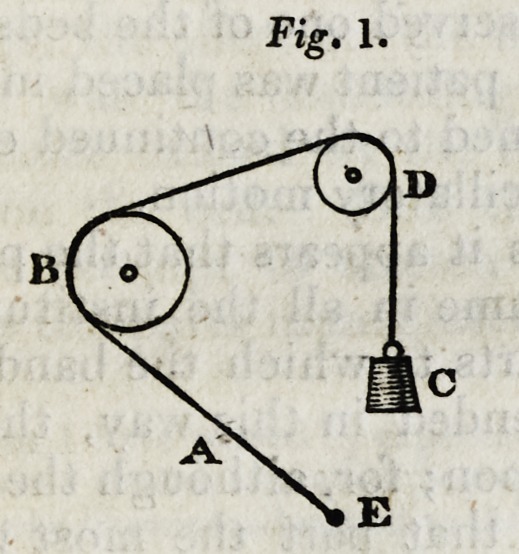


**Fig. 2. f5:**
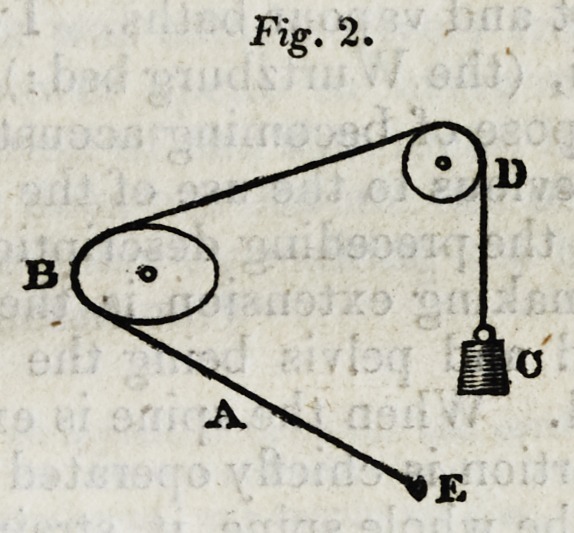


**Figure f6:**